# Correspondence: A method for determining total nitrogen...

**DOI:** 10.1155/S1463924687000397

**Published:** 1987

**Authors:** A. Fleck


					Journal of Automatic Chemistry ofClinical Laboratory Automation, Vol. 9, No. 4 (October-December 1987), p. 198
Correspondence: A method for
determining total nitrogen...
From Professor A. Fleck
I read with interest the paper by Geiger, Dennis et al. in the journal's Volume 9, Number 2, pages 72-76: 'A
method for determining to.tal nitrogen in Kjeldahl digestion solution using a centrifugal analyser'.
Without wishing to detract from the value and considerable interest ofthis application ofa centrifugal analyser,
there are a few points which it is unfortunate that the authors did not comment on or note in their paper.
The digestion method using a small proportion of a strong oxidizing agent perchloric acid seems to have been
taken from a 'continuous flow method', which was not designed for discrete digestion and analysis. There is
considerable evidence that satisfactory digestion ofeven tMrly refractory substances can be achieved without the
use ofperchloric acid (1) and it is now usually recommended that perchloric acid digestion should be avoided if
at all possible because of the hazards of explosion. There is also considerable evidence that the use of selenium
dioxide as a catalyst may lead to incomplete recovery of nitrogen (see [1]). This is also a hazard with
temperatures above 400 C if that was the temperature of the digest 1].
It seems to be a pity that the authors did not explore the possibility of using the rate of colour development to
quantitate the ammonia, preliminary observations of which have been reported by Weichselbaum et al. [2].
Finally, the authors give no evidence of the recovery of the analyte from real samples.
Thus, although the authors show that, as might be anticipated, the centrifugal analyser can be satisfactorily used
for the determination of ammonia in solution; I would strongly recommend that others wishing to use this
procedure for determining ammonia in Kjeldahl digests should carefully check on the recommended conditions
for the Kjeldahl digestion before following the details of the authors' procedure.
Department ofChemical Pathology, Charing Cross and Westminster Hospital Medical School, Fulham Palace Road, London
W6 8RF.
References
FLECK, A. Micro determination of nitrogen. Critical Reviews in Analytical Chemistry (1974), 141-154.
WEICISF.LBAtM, T. E., HAaeARTY, J. C., MARK, H. B. JNR., Analytical Chemistry, 41 (1969), 848.
FORTHCOMING PAPERS
The following are amongst the articles to the published in the Journal ofAutomatic Chemistry, Volume 10:
Instrument selection and evaluation
F. L. Mitchell
Automated processing of whole blood samples into microlitre aliquots of plasma
C. A. Burtis et al.
Robots in the clinical laboratory
L. B. Roberts
A novel approach to non-segmented flow analysis. Part 2. A prototype high-performance analyser
D. T. Malcolme-Lawes and C. Pasquini
Automated serum chloride analysis using the Apple computer
P. J. Taylor and R. A. Bouska
198

				

